# Gait and Falls in Benign Paroxysmal Positional Vertigo: A Systematic Review and Meta-analysis

**DOI:** 10.1097/NPT.0000000000000438

**Published:** 2023-03-07

**Authors:** Sara Pauwels, Laura Casters, Nele Lemkens, Winde Lemmens, Kenneth Meijer, Pieter Meyns, Raymond van de Berg, Joke Spildooren

**Affiliations:** Faculty of Rehabilitation Sciences, REVAL-Rehabilitation Research Centre, Hasselt University, Diepenbeek, Belgium (S.P., L.C., P.M., J.S.); Department of Otorhinolaryngology and Head & Neck Surgery, School for Mental Health and Neuroscience, Faculty of Health Medicine and Life Sciences, Maastricht University Medical Center+, Maastricht, the Netherlands (S.P., R.v.d.B.); Department of Otorhinolaryngology, Head and Neck Surgery, ZOL Hospital, Genk, Belgium (N.L., W.L.); and Department of Nutrition and Movement Sciences, Maastricht University, Maastricht, the Netherlands (K.M.).

**Keywords:** benign paroxysmal positional vertigo, falls, fear of falling, gait, repositioning maneuver

## Abstract

**Background and Purpose:**

Benign paroxysmal positional vertigo (BPPV) is one of the most common vestibular disorders, and is treated effectively with particle repositioning maneuvers (PRM). The aim of this study was to assess the influence of BPPV and treatment effects of PRM on gait, falls, and fear of falling.

**Methods:**

Three databases and the reference lists of included articles were systematically searched for studies comparing gait and/or falls between (1) people with BPPV (pwBPPV) and controls and (2) pre- and posttreatment with PRM. The Joanna Briggs Institute critical appraisal tools were used to assess risk of bias.

**Results:**

Twenty of the 25 included studies were suitable for meta-analysis. Quality assessment resulted in 2 studies with high risk of bias, 13 with moderate risk, and 10 with low risk. PwBPPV walked slower and demonstrated more sway during tandem walking compared with controls. PwBPPV also walked slower during head rotations. After PRM, gait velocity during level walking increased significantly, and gait became safer according to gait assessment scales. Impairments during tandem walking and walking with head rotations did not improve. The number of fallers was significantly higher for pwBPPV than for controls. After treatment, the number of falls, number of pwBPPV who fell, and fear of falling decreased.

**Discussion and Conclusions:**

BPPV increases the odds of falls and negatively impacts spatiotemporal parameters of gait. PRM improves falls, fear of falling, and gait during level walking. Additional rehabilitation might be necessary to improve gait while walking with head movements or tandem walking.

**Video Abstract available** for more insights from the authors (see the Supplemental Digital Content Video, available at: http://links.lww.com/JNPT/A421).

## INTRODUCTION

Falls are the second leading cause of unintentional injury deaths worldwide and are a major public health problem.^1^ The risk of falls increases with age and 28% to 35% of people 65 years or older experience a fall at least once a year.^2^ A fall is defined as an event that results in a person inadvertently coming to rest on the ground, floor, or other lower level.^1^,^2^

The risk of severe injuries following a fall also increases with age.^3^ Further, fear of falling (FoF) can initiate a vicious cycle of activity avoidance, functional decline, and decreased self-confidence.^4^ Cognitive impairments, medication use, environmental-related factors, gait disorders, and vestibular dysfunction are the most prevalent and significant fall risk factors in older adults.^5^,^6^

The prevalence of unidentified vestibular impairments in older adults referred to a fall clinic for nonsyncopal falls is 80%.^7^ Unfortunately, vestibular tests are rarely included in fall clinics or fall prevention programs.

Benign paroxysmal positional vertigo (BPPV) is the most commonly reported vestibular disorder.^8^ The age of onset of BPPV is most often between 50 and 70 years,^8^ and affects more women than men (2.4:1).^9^ BPPV is caused by dislodged otoconia from the utricular macula in the inner ear. When otoconia migrate into one of the semicircular canals or attach to the cupula of the ampullae, deflection of the cupula occurs. As a result, BPPV causes positional nystagmus and short repeated episodes of rotational vertigo induced by head position changes in the plane of the semicircular canals. People with BPPV (pwBPPV) can experience symptoms of vertigo, imbalance, and nausea.^10^ Moreover, due to its higher prevalence in women and its association with osteoporosis, pwBPPV have a 1.14-fold elevated risk of fractures from falls compared with those without BPPV.^11^

BPPV is diagnosed when nystagmus is provoked during positional tests, such as Dix-Hallpike and supine roll, depending on the involved canal.^12^,^13^ It can be cured using noninvasive treatment approaches called particle repositioning maneuvers (PRM), such as the Epley maneuver,^14^ Sémont maneuver,^14^ or barbeque roll maneuver.^15^ With PRM, the therapist aims to relocate the dislodged otoconia by performing consecutive movements in the plane of the affected semicircular canal to relieve the symptoms of BPPV.^16^

The aim of this systematic review and meta-analysis was to explore the impact of BPPV and the treatment effects of PRM on falls and FoF. Since gait disorders are significant fall risk predictors, the impact of BPPV and the treatment effect of PRM on gait are also discussed.

## METHODS

### Protocol and Registration

This study was conducted according to the preferred reporting items for systematic reviews and meta-analysis (PRISMA) protocol (www.crd.york.ac.uk/prospero; registration no. CRD42021261848).

### Literature Search

In June 2021 and February 2022, PubMed, Web of Science, and Scopus were systematically searched by 2 independent reviewers (S.P. and L.C.). References of the included articles were screened to ensure that no relevant articles were missed. Search strategies were based on synonyms for the keywords “BPPV,” “gait,” “falls,” and “FoF” (see Supplemental Digital Content 1, available at: http://links.lww.com/JNPT/A419, which demonstrates search strings). No filters were applied.

Articles written in English, Dutch, or French with a cohort, case-control, or controlled study design were considered relevant. To be included, a comparison of adults with BPPV (≥18 years) and controls or a pre- and posttreatment comparison with PRM needed to be made for at least one of the following outcomes: measures of spatiotemporal parameters of gait, events of falling, and/or FoF.

Exclusion criteria were (i) the presence of BPPV in combination with other disorders (eg, Parkinson disease) that could interfere with the outcome measures, (ii) self-evaluation of gait, (iii) the use of (or combination of PRM with) other treatments (eg, vestibular rehabilitation), and (iv) conference proceedings/reposts, editorials, letters, case studies/series, (systematic) reviews, and meta-analyses. In the case of multiple publications on the same subject sample and outcome measure, only the study with the largest sample size was retrieved for inclusion to avoid overrepresentation of these subjects.

### Quality Assessment

The Joanna Briggs Institute (JBI) critical appraisal tools^17^ were used to identify risk of bias by 2 independent researchers (S.P. and L.C.). The checklist for case-control studies or quasi-experimental studies was used to evaluate the impact of BPPV or the treatment effect of PRM, respectively. Articles were graded as “low risk of bias” (≥70% yes score), “moderate risk of bias” (50%-69% yes score), or “high risk of bias” (<50% yes score).

The method for rating was standardized, and the results were discussed in a consensus meeting. If a consensus was not reached, a third researcher (J.S.) was consulted.

### Data Extraction

General population characteristics (number of participants per group, mean age, and standard deviation [SD], age range, and sex distribution) and specific characteristics for patient groups and treatment (semicircular canal affected, executed PRM, and follow-up after treatment) were collected. Articles were classified as “pwBPPV versus (vs) control” or “treatment effect of PRM” for studies comparing pwBPPV to controls and/or measures before and after PRM, respectively.

Results on gait were classified according to the task (eg, level walking and Timed Up & Go [TUG]^18^), and sensory alteration applied. When only the total score of a scale for gait assessment was reported (eg, Dynamic Gait Index (DGI)^19^ or Functional Gait Assessment [FGA]^20^), the derived data were classified as “gait assessment scale.” Results on falls were classified as the number of falls (ie, number of falls over a defined period), fall incidence (number of people fallen in a defined period), and FoF (ie, Falls Efficacy Scale–International [FES-I]^21^ or Activities-specific Balance Confidence [ABC] scale^22^). If multiple measurements post-treatment were reported, data from the earliest measurement were derived for the meta-analysis.

Numeric values (mean and SD) for each outcome were extracted. When median and range were reported, mean variance and SD were estimated using the method of Hozo et al.^23^

### Data Synthesis and Analysis

If an outcome measure was discussed in 3 or more articles, a meta-analysis of the raw data was executed^24^ using Review Manager (Version 5.4.1, The Cochrane Collaboration, 2020). To conduct the meta-analysis, the means, SDs, and number of participants in each group were used. For continuous variables, standardized mean differences (SMDs) were calculated with a random-effects model. For dichotomous outcome measures, odds ratios were calculated using the Mantel-Haenszel method with a random-effects model.

Confidence intervals (CIs) were set at 95%. A significance level of *P* < 0.05 was applied for all outcome measures. Heterogeneity between the publications was measured using the Higgins *I*² statistic^25^ and was classified as low (<50%), moderate (<75%), or high (>75%). When no raw data were available in the article, the authors of the corresponding article were contacted by email. Outcomes that could not be included in a meta-analysis were described.

## RESULTS

### Literature Search

**Figure 1:**
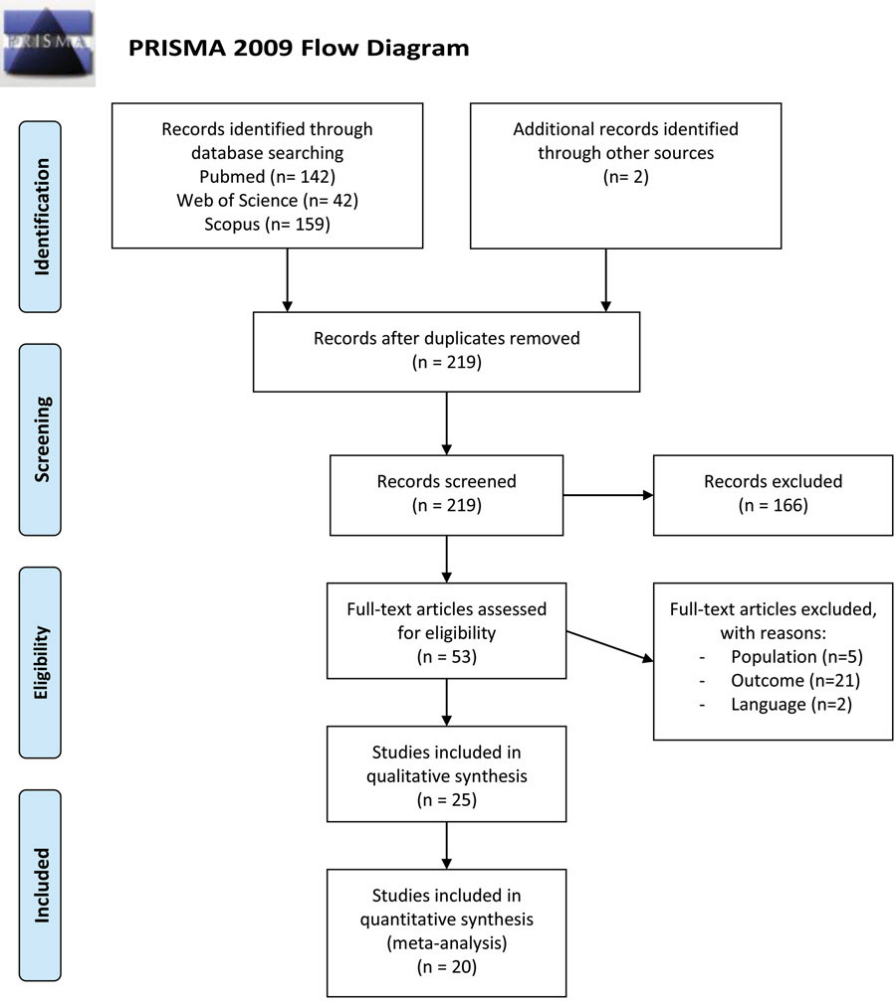
Flowchart of the selection process. From Moher et al.^51^ This figure is available in color online (www.jnpt.org).

The systematic search resulted in 219 unique hits. Of these 219 publications, 25 met the selection criteria. Data extracted from 20 articles^26^–^45^ could be pooled in meta-analyses, and 5 additional studies^46^–^50^ were only included for descriptive data. An overview of the literature search is shown in Figure 1.^51^

### Risk of Bias in Individual Studies

Ten studies were assessed with the JBI critical appraisal checklist for case-control studies.^28^–^30^,^36^,^37^,^44^ Two studies were classified as high,^28^,^30^ 6 as moderate,^33^,^36^,^39^,^42^,^44^,^49^ and 2 as low risk^29^,^37^ of bias. The 2 studies with a high risk of bias had a cross-sectional design, screening for BPPV in a cohort.^28^,^30^ Comparable cases and controls were included in 6 studies.^33^,^36^,^37^,^39^,^44^,^49^ In 1 study, the presence of nystagmus was checked with the use of defocusing goggles (eg, Frenzel, videonystagmography), which is believed to improve diagnostic accuracy and a valid measurement of the exposure.^29^ In 2 studies, it was unclear whether the presence of BPPV was checked in the control group.^36^,^39^ All studies used appropriate statistical analyses.

Fifteen studies were assessed with the JBI critical appraisal checklist for quasi-experimental studies.^26^,^27^,^31^,^32^,^34^,^35^,^38^,^40^,^41^,^43^,^45^–^48^,^50^ None of the studies were classified as having a high risk of bias. Seven studies were identified as moderate^34^,^35^,^43^,^45^–^47^,^50^ and 8 as low risk of bias.^26^,^27^,^31^,^32^,^38^,^40^,^41^,^48^ Eight studies had a single-group pre-/posttest design.^26^,^34^,^35^,^43^,^45^–^47^,^50^ Therefore, the differences in treatment/care or ways to measure the outcomes between groups was not applicable.^52^ In all studies, the cause and effect was clear and follow-up was completed. In 3 studies, a statistical power analysis was performed.^26^,^27^,^40^ Supplemental Digital Content 2 (available at: http://links.lww.com/JNPT/A420) provides an overview of the risk of bias assessment for case-control and quasi-experimental studies.

### Study and Population Characteristics

In total, 1016 pwBPPV with a mean age ranging from 51^26^ to 83^37^ years and 1581 controls with a mean age from 48^27^ to 83^37^ years were included. In 13 studies, only BPPV of the posterior canal was included,^26^,^31^–^38^,^40^,^41^,^43^,^44^ while 8 studies also included the lateral and/or anterior canal.^27^,^29^,^39^,^45^–^48^,^50^ In 4 studies, the affected canal was not specified.^28^,^30^,^42^,^49^

A total of 517 pwBPPV received treatment with PRM. Posterior canal BPPV was treated with the Epley,^26^,^32^,^34^,^40^,^46^–^48^,^50^ modified Epley,^27^,^31^,^35^,^38^ or Sémont^43^ maneuver. Involvement of the lateral canal was treated with the barbeque roll^27^,^45^,^46^,^48^,^50^ or Gufoni maneuver.^45^ The Epley,^46^ reversed Epley,^47^ and Rahko's maneuver^48^ were applied to treat anterior canal involvement (Table 1). The time of earliest reevaluation after treatment ranged from the time after confirmed resolution of nystagmus for FoF,^48^ until 12 months after resolution for falls.^53^

**Table 1:** Study and Population Characteristics

Study		BPPV			Treatment		Control Group	
Author	Design	n (F/M)	Canal (n)	Age, Mean ± SD, y	PRM	Follow-up	n (F/M)	Age, Mean ± SD, y
Balci and Akdal^26^	Prospective	57 (41/16)	PC	51.45 ± 13.29	Epley	1 wk 1 mo		
Çelebisoy et al^27^	Prospective	44	PC (32) LC (12)	55 (range 32-77) 55.6 (range 39-74)	Modified Epley BBQ	1 wk/2 wk	50	48.3 (range 27-70)
Chang et al^38^	Randomized controlled trial	13 (7/6)	PC	53.93 ± 9.97	Modified Epley	2 wk/4 wk		
Cohen et al^39^	Prospective	21 (11/10)	PC + LC (13)	58.8 ± 11.7			61 (30/31)	49.6 ± 16.0
Cohen-Shwartz et al^40^	Prospective	32 (25/7)	PC	64.3 ± 6.4	Epley	1 wk	15 (9/6)	63.5 ± 7.1
D'Silva et al^41^	Prospective	34 (29/5)	PC	58.85 ± 10.65	Unclear	7-10 d		
Ganança et al^46^	Retrospective	121 (71/50)	PC (100) LC (16) AC (4)	Range 65-89	Epley BBQ	12 mo		
Huang et al^42^	Retrospective	255 (178/77)	Unclear	65.4 ± 12.0			295 (159/136)	59.1 ± 17.7
Jumani and Powell^47^	Retrospective	40 (27/13)	PC (39) AC (1)	>65 y	Epley Reverse Epley	6 mo		
Jung et al^48^	Randomized controlled trial	34 (21/13)	PC (14) LC (12) AC (3)	53	Epley Rahko's BBQ	After confirming resolution of nystagmus		
Kollén et al^43^	Prospective	17 (13/4)	PC	52 (range: 31-66)	Sémont	1 mo/6 mo/12 mo		
Kollén et al^44^	Prospective	63 (46/17)	PC	75			508 (286/222)	75
Lim et al^45^	Prospective	33 (23/11)	PC (24) LC (10)	60.20 ± 11.94	Epley BBQ Gufoni	Mean: 8.73 d (SD: 5.94)		
Lindell et al^28^	Prospective, cross-sectional	11 (8/3)	Unclear	75			403 (224/179)	75
Lindell et al^29^	Prospective	15 (14/1)	PC (10) LC (5)	79 ± 3.8			40 (38/2)	78 ± 4.5
Hawke et al^49^	Prospective	18	Unclear	69 ± 13			16	69 ± 13
Oghalai et al^30^	Cross-sectional	9	Unclear	74 ± 1			91	Unclear
Se To et al^31^	Randomized controlled trial	14 (13/1)	PC	54.36 ± 8.55	Modified Epley	4 wk/6 wk		
Ribeiro et al^32^	Randomized controlled trial	7 (5/2)	PC	71.75 ± 3.15	Epley	1 wk/5 wk/ 9 wk/13 wk		
Roberts et al^33^	Case-control	15 (10/5)	PC	55.6 ± 9.8			15 (7/8)	48.5 ± 9.8
Maslovara et al^50^	Prospective clinical trial	81 (59/22)	PC (79) LC (2)	60.1 ± 12.1	Epley BBQ	1 wk after cure		
Silva et al^34^	Prospective, quasi-experimental	14 (11/3)	PC	71 ± 4.05	Epley	1 wk		
Vaz et al^35^	Prospective clinical	30 (28/2)	PC	70.10 ± 7.00	Modified Epley	1 wk		
Zhang et al^36^	Prospective	27 (16/11)	PC	56.5 ± 13.1			27 (21/6)	56.1 ± 10.8
Zur et al^37^	Prospective	11	PC	83 ± 5			60	83 ± 5

Abbreviations: AC, anterior semicircular canal BPPV; BBQ, barbeque roll maneuver; BPPV, benign paroxysmal positional vertigo; F, female; LC, lateral semicircular canal BPPV; M, male; PC, posterior semicircular canal BPPV; PRM, particle repositioning maneuver; SD, standard deviation.

### Results for Gait

The results on spatiotemporal parameters during different gait tasks are summarized in Table 2.

**Table 2:** Results on Spatiotemporal Parameters During Different Gait Tasks

	Without Sensory Alterations	Alterations of Vision	Head Rotations	Tandem Walking	Dual Task
Gait velocity					
pwBPPV vs control	↘ *P* < 0.0001; SMD = −0.75^a^	↘	↘	↘	↘
Treatment effect	↗ *P* = 0.001; SMD = −0.51^a^	/	=	= older pwBPPV ↗ younger pwBPPV	/
Cadence					
pwBPPV vs control	↘ *P* = 0.02; SMD = −0.48^a^	/	=		/
Treatment effect	↗	/	/		/
Step/stride length					
pwBPPV vs control	↘ *P* = 0.005; SMD = −0.54^a^	/	↘		/
Treatment effect	↗ *P* = 0.001; SMD = −0.51^a^	/	/		/
Sway velocity					
pwBPPV vs control	/	/	/	=	/
Treatment effect	/	/	/	= *P* = 0.22; SMD = 0.20^a^	/

Abbreviations*:* pwBPPV, people with benign paroxysmal positional vertigo; SMD, standardized mean difference; **↘**, significantly decreased in people with benign paroxysmal positional vertigo; **↗**, significantly improved after treatment with particle repositioning maneuvers; =, no significant difference; **/,** no literature available.

^a^*P* values and SMD of meta-analysis.

#### Gait Assessment Scales

None of the included studies used a gait assessment scale to measure the *impact of BPPV* on gait.

**Figure 2:**
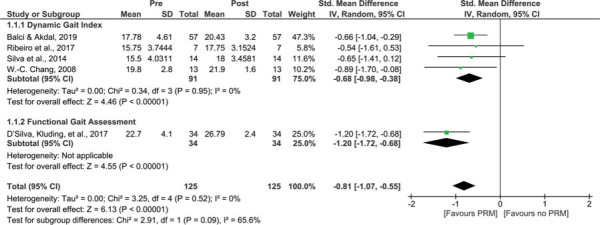
Treatment effect of PRM on end scores of gait assessment scales. A comparison of end scores of gait assessment scales of people with BPPV before treatment with PRM (pre) and after treatment with PRM (post). A significant result is visualized by the diamond shape not crossing the central vertical line. BPPV, benign paroxysmal positional vertigo; CI, confidence interval; IV, inversed variance; PRM, particle repositioning maneuvers; Std, standardized. This figure is available in color online (www.jnpt.org).

In 5 studies, *the treatment effect of PRM* on gait was evaluated using the DGI^26^,^32^,^34^,^38^ or the FGA.^41^ The DGI uses a 4-point scale to assess gait during 8 different tasks. The FGA includes 7 tasks of the original DGI in combination with 3 additional items. Meta-analysis revealed a significant improvement after treatment (*P* < 0.001; SMD = −0.81, 95% CI −1.07 to −0.55), without heterogeneity (*I*^2^ = 0%) (Figure 2).

#### Timed Up & Go

“Time” to perform the TUG was *compared between pwBPPV and controls* in 2 studies.^29^,^40^ PwBPPV take significantly longer to perform a TUG compared with controls (*P* < 0.001). When pwBPPV were compared with older adults with complaints of dizziness (but without vestibular disorder), the time to perform the TUG did not differ significantly (*P* = 0.6).^29^ No significant differences were found in turn characteristics during the TUG between pwBPPV and controls.^40^

**Figure 3:**
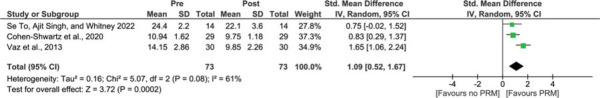
Treatment effect of PRM on time (seconds) to perform Timed Up & Go. A comparison of time to perform Timed Up & Go of people with BPPV before treatment with PRM (pre) and after treatment with PRM (post). A significant result is visualized by the diamond shape not crossing the central vertical line. BPPV, benign paroxysmal positional vertigo; CI, confidence interval; IV, inversed variance; PRM, particle repositioning maneuvers; Std, standardized. This figure is available in color online (www.jnpt.org).

*The treatment effect* on “time” to perform the TUG was investigated in 3 studies.^31^,^35^,^40^ After PRM, pwBPPV performed the TUG significantly faster than before PRM (*P* < 0.001; SMD = 1.09, 95% CI 0.52 to 1.67) (Figure 3). Heterogeneity in the meta-analysis was moderate (*I*^2^ = 61%). Significant improvements were found for turn velocity (*P* = 0.007), but not for turn duration and steps.^40^

#### Walking Without Sensory Alterations

Differences in spatiotemporal parameters between pwBPPV and controls were assessed in 7 studies.^28^,^29^,^33^,^36^,^39^,^40^,^44^

**Figure 4:**
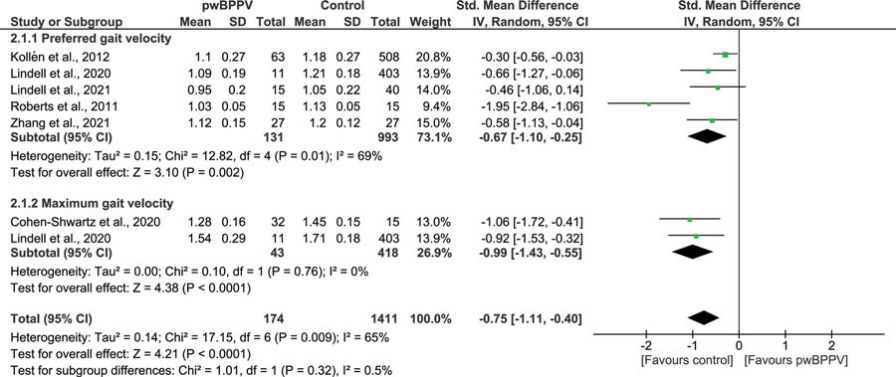
Impact of BPPV on gait velocity (m/s). A comparison of gait velocity (m/s) between people with BPPV and controls. A significant result is visualized by the diamond shape not crossing the central vertical line. BPPV, benign paroxysmal positional vertigo; CI, confidence interval; IV, inversed variance; PwBPPV, people with BPPV; Std, standardized. This figure is available in color online (www.jnpt.org).

In 6 studies, “gait velocity” was assessed during preferred^28^,^29^,^33^,^36^,^40^,^44^ or maximum gait velocity.^28^,^40^ PwBPPV walked significantly slower compared with controls. Meta-analysis revealed an SMD of −0.75 (*P* < 0.001; 95% CI −1.11 to −0.40), with significant (*P* = 0.009) moderate heterogeneity (*I*^2^ = 65%) (Figure 4). When the study with a high risk of bias was excluded from the meta-analysis, the results were still significant.^28^

**Figure 5:**
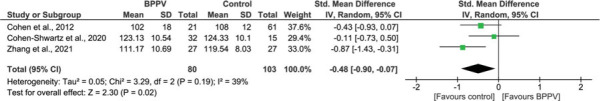
Impact of BPPV on cadence (steps/min). A comparison of cadence (steps/min) between people with BPPV and controls. A significant result is visualized by the diamond shape not crossing the central vertical line. BPPV, benign paroxysmal positional vertigo; CI, confidence interval; IV, inversed variance; PwBPPV, people with BPPV; Std, standardized. This figure is available in color online (www.jnpt.org).

According to a meta-analysis of 3 studies,^36^,^39^,^40^ “cadence” was significantly lower in pwBPPV compared with controls (*P* = 0.02; SMD = −0.48, 95% CI −0.90 to −0.07), with low heterogeneity (*I*^2^ = 39%) (Figure 5).

**Figure 6:**
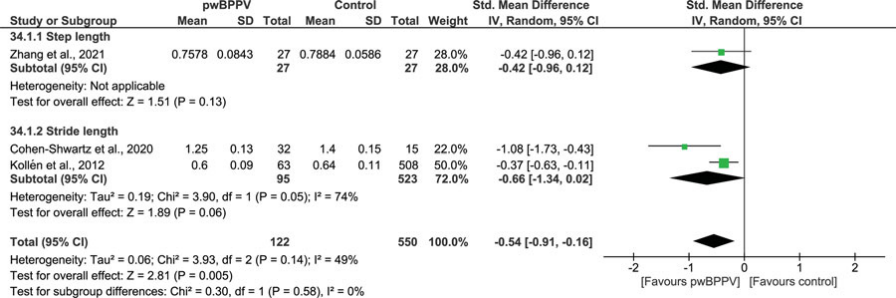
Impact of BPPV on step and stride length (m). A comparison of step and stride length (m) between people with BPPV and controls. A significant result is visualized by the diamond shape not crossing the central vertical line. BPPV, benign paroxysmal positional vertigo; CI, confidence interval; IV, inversed variance; PwBPPV, people with BPPV; Std, standardized. This figure is available in color online (www.jnpt.org).

Meta-analysis of 3 studies^36^,^40^,^44^ also revealed that “stride length”^40^,^44^/“step length”^36^ were significantly lower in pwBPPV (*P* = 0.005; SMD = −0.54, 95% CI −0.91 to −0.16), with low heterogeneity (*I*^2^ = 49%) (Figure 6).

**Figure 7:**
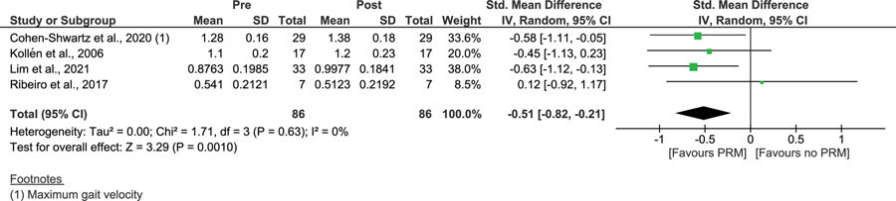
Treatment effect of PRM on gait velocity (m/s). A comparison of gait velocity (m/s) of people with BPPV before treatment with PRM (pre) and after treatment with PRM (post). A significant result is visualized by the diamond shape not crossing the central vertical line. BPPV, benign paroxysmal positional vertigo; CI, confidence interval; IV, inversed variance; PRM, particle repositioning maneuvers; Std, standardized. This figure is available in color online (www.jnpt.org).

In 4 studies, the *treatment effect of PRM* on spatiotemporal parameters was assessed during walking without sensory alterations.^34^,^40^,^43^,^45^ For “gait velocity,” significant improvements were found after PRM with an SMD of −0.51 (*P* = 0.001; 95% CI −0.82 to −0.21), without heterogeneity (*I*^2^ = 0%) (Figure 7).^34^,^40^,^43^,^45^

**Figure 8:**
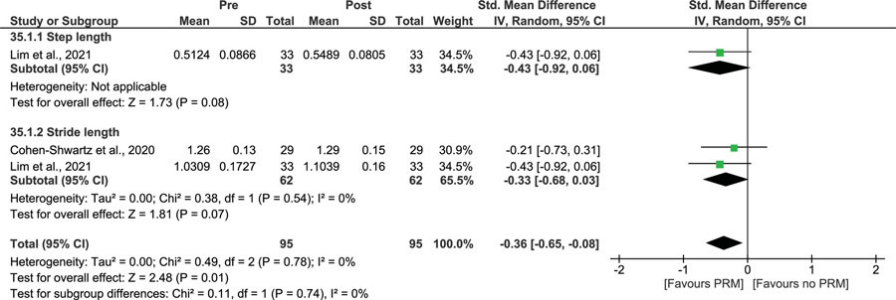
Treatment effect of PRM on step and stride length (m). A comparison of step and stride length (m) of people with BPPV before treatment with PRM (pre) and after treatment with PRM (post). A significant result is visualized by the diamond shape not crossing the central vertical line. BPPV, benign paroxysmal positional vertigo; CI, confidence interval; IV, inversed variance; PRM, particle repositioning maneuvers; Std, standardized. This figure is available in color online (www.jnpt.org).

Meta-analysis also revealed significant improvements for “step/stride length” (*P* = 0.01; SMD = −0.36, 95% CI −0.65 to −0.08), without heterogeneity (*I*^2^ = 0%) (Figure 8).^40^,^45^

Other spatiotemporal parameters were reported in 2 studies.^40^,^45^ Significant improvements were found for “cadence,” but results on “double support time/phase” were conflicting.^40^,^45^ In one study, significant improvements were also found for “stance time” and “step characteristics (width and time),” but not for “single support time,”^45^ “swing time,” or “base of support.”^45^

#### Walking With Alterations in Vision

In 1 study, “gait velocity” was assessed during walking with eyes closed. PwBPPV walked significantly slower with eyes closed *compared with controls* (*P* < 0.001).^33^ None of the included studies assessed the *treatment effect of PRM* on spatiotemporal parameters during walking with alterations in vision.

#### Walking With Head Movements

The *impact of BPPV* on walking with head movements was assessed in 3 studies.^33^,^39^,^44^

During both walking with vertical^33^ and horizontal head movements,^44^ pwBPPV walked significantly slower compared with controls.

A significant decrease in “stride length” (*P* < 0.001) when walking with horizontal head movements was also found.^44^ For “cadence” during walking with horizontal head movements, no significant differences were found (*P* = 1.0).^39^

One study measured the *treatment effect of PRM* on walking speed during walking with head movements. One month after treatment, “gait velocity” did not significantly improve during walking with horizontal and vertical head movements.^43^ Six and 12 months after treatment, gait velocity did improve.

#### Tandem Walking

In 2 studies, tandem walking was compared between *pwBPPV and controls*. PwBPPV walked significantly slower during tandem walking but did not present with more “end sway of their center of gravity” or “fewer consecutive steps” compared with controls.^27^,^39^

**Figure 9:**
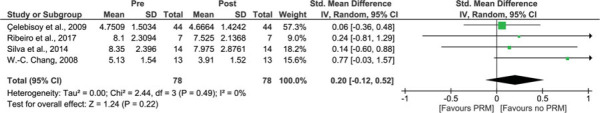
Treatment effect of PRM on center of gravity sway velocity during tandem walking (°/s). A comparison of center of gravity sway velocity during tandem walking (°/s) of people with BPPV before treatment with PRM (pre) and after treatment with PRM (post). A significant result is visualized by the diamond shape not crossing the central vertical line. BPPV, benign paroxysmal positional vertigo; CI, confidence interval; IV, inversed variance; PRM, particle repositioning maneuvers; Std, standardized. This figure is available in color online (www.jnpt.org).

In 4 studies, “sway velocity of center of gravity” of tandem walking was compared *pre- and post-treatment*.^27^,^32^,^34^,^38^ Meta-analyses resulted in an SMD of 0.20 (95% CI −0.12 to 0.52), but the difference (*P* = 0.22) was not significant. There was no heterogeneity in the pooled sample (*I*^2^ = 0%) (Figure 9).

After treatment, “tandem walking speed” improved in younger pwBPPV,^53^ but not in older pwBPPV.^34^

#### Walking With Cognitive Dual Tasks

In 1 study, “gait velocity” of walking during a cognitive dual task was compared *between pwBPPV and controls*. PwBPPV walked significantly slower during this dual task compared to controls.^33^ Gait velocity was more affected during dual tasking than in normal level walking, as pwBPPV walked 8.5% slower than controls during normal level walking, but 19% slower during dual tasking.^33^

None of the included studies assessed the *treatment effect of PRM* on spatiotemporal parameters during walking with cognitive dual tasks.

### Results on Falls

#### Incidence of Falls

**Figure 10:**
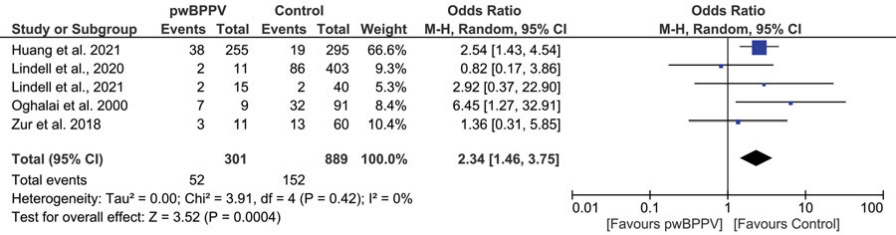
Impact of BPPV on fall incidence. A comparison of fall incidence between people with BPPV and controls. A significant result is visualized by the diamond shape not crossing the central vertical line. BPPV, benign paroxysmal positional vertigo; CI, confidence interval; M-H, Mantel-Haenszel; PwBPPV, people with BPPV. This figure is available in color online (www.jnpt.org).

In 5 studies, *pwBPPV and controls* were asked whether they experienced falls in a previously defined period.^28^–^30^,^37^,^42^ With an odds ratio of 2.34, pwBPPV had a significant increased odds of falling compared with controls (*P* < 0.001; 95% CI 1.46 to 3.75), without heterogeneity (*I*^2^ = 0%) (Figure 10). Exclusion of the studies with a high risk of bias did not affect the significance or odds ratio.^28^,^30^

In 1 study, the number of pwBPPV who fell was significantly reduced after PRM.^47^

#### Number of Falls

The number of falls reported in the previous year was studied in 2 articles by Lindell et al.^28^,^29^ In a population-based cohort study of 75 year olds, pwBPPV reported significantly more falls *compared with controls* (*P* = 0.013).^28^ When pwBPPV were compared with older adults with complaints of dizziness (but without vestibular disorder), the number of falls did not differ significantly (*P* = 0.9).^29^

The *treatment effect of PRM* on the reported number of falls was retrospectively reviewed in 2 studies. In both studies, the number of falls (at 6 and 12 months) was significantly reduced after PRM.^46^,^47^

#### Fear of Falling

The *impact of BPPV* on FoF was measured in 1 study^49^ with the FES-I. The FES-I assesses a person's concern about falling during a range of physical and social activities. PwBPPV and controls were recruited from a rehabilitation center for older people with a high fall risk. No significant difference was found in the FES-I (*P* = 0.481) between pwBPPV (mean score 36.7) and controls (mean score 39.4).

Three articles investigated the *treatment effect of PRM* on FoF.^31^,^48^,^50^ In all 3, the ABC scale was used: a 16-item scale that questions balance confidence during various activities. A significant improvement in ABC scores after PRM was found in all 3 studies. Data could not be pooled, as Jung et al^48^ did not publish SDs. The weighted mean ABC score changed from 60.5% before treatment to 83.7% after treatment with PRM.

## DISCUSSION

This study aimed to explore the impact of BPPV and treatment effect of PRM on gait, falls, and FoF. BPPV negatively affects the spatiotemporal parameters of gait during all different gait tasks. After treatment with PRM, pwBPPV walked significantly faster during normal level walking and performed better on gait assessment scales. Furthermore, treatment decreased their fall incidence, number of falls, and FoF.

During all different gait tasks, pwBPPV walked significantly slower than controls. For normal level walking, PRM improved gait velocity, cadence, and step/stride length. However, during walking with head movements, gait velocity and cadence improved only at 6 and 12 months after treatment. Despite the improvement in FoF, 1 month after treatment, pwBPPV may still experience fear of provoking symptoms with head movements or may still need to rely more on other sensory systems (eg, vision), causing them to walk more slowly.

Tandem walking did not improve after PRM, nor during further follow-up. A positive treatment effect of PRM on tandem walking was found only in pwBPPV receiving additional vestibular rehabilitation.^32^ The results on walking with head movements and tandem walking might suggest that additional (vestibular and/or gait) rehabilitation, or more time, is necessary to recover their gait during more challenging situations (which are more in line with real-life settings). Literature on the treatment effect on gait parameters during walking with visual alterations or with cognitive dual tasks was not available.

Several improvements were not only significantly different but also exceeded the minimal clinical important difference (MCID). Time to perform the TUG was reduced by 2.69 seconds (MCID = 1.2 seconds)^54^ and the DGI improved by 2.40 points (MCID = 1.9 points).^55^

With a significant odds ratio of 2.34 on fall incidence, pwBPPV are more likely to fall than their peers. This might also explain why unrecognized BPPV is highly prevalent, even up to 54% in older adults referred to fall clinics.^49^,^56^

The lack of significant difference on FoF between pwBPPV and controls might be explained by the study sample (ie, older adults from a falls clinic), as both groups have an increased risk of falling. Nevertheless, both the FES-I and ABC scale scores of pwBPPV pre-treatment indicated an increased FoF^21^ and moderate level of physical functioning,^22^ respectively. After treatment, the improved ABC scores correlated with a high level of physical functioning and a decreased risk of falling.^57^

The prevalence of undiagnosed BPPV in older adults ranges from 9%^30^ to 11%^44^ in the community-dwelling, and 11.3% in nursing homes.^58^ Besides an increased risk of falls and fractures, undiagnosed BPPV can lead to a reduced quality of life and increased feelings of depression.^30^ Older pwBPPV often present with more vague symptoms of general dizziness and instability rather than classic symptoms of vertigo, which are often considered a normal part of aging.^59^,^60^

The cost to arrive at a diagnosis of BPPV is estimated at $2000,^61^ and is associated with multiple consultations and unnecessary laboratory testing. This budget might even be an underestimation since it does not account for the consequences of the increased odds of falling in pwBPPV.

Our results highlight the importance of including positional tests for BPPV in the diagnostic process of older adults with an increased fall risk, particularly those with complaints of dizziness. Unfortunately, despite the noninvasive diagnostic and treatment maneuvers, BPPV often seems overlooked as a possible diagnosis in older adults with an increased fall risk. More research that charts specific features and enhances the early detection of BPPV in people with increased fall risk is necessary. Both diagnosis and treatment for BPPV are low cost and can be performed by trained primary care providers. As the incidence of BPPV increases with age and the global population ages, this can provide both a clinical and socioeconomic win.

### Limitations

Some limitations should be acknowledged in this study. Until now, only one study has reviewed fall incidence prospectively.^37^ The other studies evaluated fall incidence retrospectively. Prospective research on falls is considered the best method to investigate falls, as retrospective research can have recall bias.^62^ Second, no comparison was made between PRM and other treatments or placebo to assess the treatment effect of PRM, as literature on this is limited.

Finally, there was noticeable heterogeneity within and between the included studies. Studies assessing gait in pwBPPV included broad age ranges, whereas studies assessing falls in pwBPPV mainly focused on older adults. The defined period for questions regarding falls ranged from 90 days to 2 years, which may have further increased recall bias.

Additionally, small sample sizes resulted in small meta-analyses. Despite this, only one meta-analysis reported significant, moderate heterogeneity.

This is the first systematic review of gait, falls, and FoF in pwBPPV. Two independent researchers performed the study selection in 3 electronic databases and through the reference lists of the included articles. In addition to English, articles in Dutch and French were also included. A detailed methodological quality assessment was also carried out by 3 independent reviewers. Only 2 of the included studies were identified as having a high risk of bias, possibly because of their designs.

Future research on this topic should prospectively assess the impact of BPPV and treatment effect of PRM on falls, FoF, and gait with sensory alterations. Sufficient sample sizes with a clear differentiation between age groups should be included. Also, after treatment, a comparison should be made between pwBPPV and age-matched controls to determine whether gait is normalized after treatment.

## CONCLUSIONS

Our results reveal that BPPV has a negative impact on gait and significantly increases the odds of falling. The gold standard, noninvasive treatment with PRM improves falls, FoF, and gait during normal level walking. Additional rehabilitation might be necessary to improve gait while walking with head movements or tandem walking. More research is necessary to identify BPPV and improve diagnosis in people who are at risk of falling. Nevertheless, greater awareness of BPPV and faster initiation of treatment may in itself prevent devastating falls.

## Supplementary Material
