# Supplementary material for: Gait and Falls in Benign Paroxysmal Positional Vertigo: A Systematic Review and Meta-analysis
Source: J Neurol Phys Ther. 2023 Mar 7;47(3):127–38. doi: 10.1097/NPT.0000000000000438 (PMC10521788; doi:10.1097/NPT.0000000000000438)
Supplement: Supplementary file 1 [file jnpt-47-127-s001.pdf]

**Supplemental Digital Content 1**

Search string

*Pubmed*

("Benign Paroxysmal Positional Vertigo"[MeSH Terms] OR "Benign Paroxysmal Positional Vertigo"[Title/Abstract] OR "BPPV"[Title/Abstract]) AND ("Gait"[MeSH Terms] OR "Gait"[Title/Abstract] OR "gait analyses"[Title/Abstract] OR "Walking"[MeSH Terms] OR "walk*"[Title/Abstract] OR "walking speed"[Title/Abstract] OR "walking speed"[MeSH Terms] OR "Locomotion"[Title/Abstract] OR "gait stability"[Title/Abstract] OR ("Falls"[Title/Abstract] OR "Falling"[Title/Abstract] OR ("accidental falls"[MeSH Terms] OR "accidental falls"[Title/Abstract] OR "fear of falling"[Title/Abstract] OR "falls efficacy scale"[Title/Abstract] OR "activities specific balance confidence scale"[Title/Abstract])))

*Web of Science*

TS= ("Benign Paroxysmal Positioning Vertigo" OR "BPPV") AND (TS= ("Gait" OR "Walking" OR "Walking Speed" OR "Gait Stability" OR "Gait analyses") OR TS=("Fall" OR "Falls" OR "Falling" OR "Accidental Fall") OR TS= ("fear of falling" OR "falls efficacy scale" OR "activities specific balance confidence scale"))

*Scopus*

( TITLE-ABS-KEY ( ( "Benign Paroxysmal Positioning Vertigo" OR "BPPV" ) ) AND TITLE-ABS-KEY ( ( ( "Gait" OR "Walking" OR "Walking Speed" OR "Gait Stability" OR "Gait analyses" ) ) ) OR TITLE-ABS-KEY ( ( "Fall" OR "Falls" OR "Falling" OR "Accidental Fall" ) ) OR TITLE-ABS-KEY ( ( "fear of falling" OR "falls efficacy scale" OR "activities specific balance confidence scale" ) ) )
