# Supplementary material for: Gait and Falls in Benign Paroxysmal Positional Vertigo: A Systematic Review and Meta-analysis
Source: J Neurol Phys Ther. 2023 Mar 7;47(3):127–38. doi: 10.1097/NPT.0000000000000438 (PMC10521788; doi:10.1097/NPT.0000000000000438)
Supplement: Supplementary file 2 [file jnpt-47-127-s002.docx]

**Supplemental Digital Content 2**

Risk of bias assessment of case-control studies

| Author | Q1 | Q2 | Q3 | Q4 | Q5 | Q6 | Q7 | Q8 | Q9 | Q10 | Risk of bias |
| --- | --- | --- | --- | --- | --- | --- | --- | --- | --- | --- | --- |
| Cohen et al., 2011 | Y | N | Y | N | U | N | N | Y | Y | Y | **Moderate** |
| Huang et al. 2021 | Y | Y | Y | N | Y | N | N | Y | Y | Y | **Low** |
| Kollén et al., 2012 | U | N | Y | N | Y | Y | Y | Y | U | Y | **Moderate** |
| Lindell et al., 2021 | Y | N | Y | Y | Y | N | N | Y | Y | Y | **Low** |
| Lindell et al., 2020 | N | N | Y | N | Y | N | N | Y | U | Y | **High** |
| LJ, CJ, and JV 2021 | Y | N | U | N | Y | N | N | Y | U | Y | **Moderate** |
| Oghalai et al. 2000 | N | N | N | N | Y | N | N | Y | U | Y | **High** |
| Roberts et al., 2011 | Y | U | U | N | Y | N | Y | Y | U | Y | **Moderate** |
| Zhang et al., 2021 | Y | Y | U | N | U | Y | Y | Y | U | Y | **Moderate** |
| Zur et al. 2018 | Y | Y | Y | N | Y | N | N | Y | Y | Y | **Low** |

Abbreviations: Y, yes; N, no; U, Unclear
Q1: Were the groups comparable other than the presence of the disease in cases or absence of disease in controls?
Q2: Were cases and controls matched appropriately?
Q3: Were the same criteria used for identification of cases and controls?
Q4: Was exposure measured in a standard, valid and reliable way?
Q5: Was exposure measured in the same way for cases and controls?
Q6: Were confounding factors identified?
Q7: Were strategies to deal with confounding factors stated?
Q8: Were outcomes assessed in a standard, valid and reliable way for cases and controls?
Q9: Was the exposure period of interest long enough to be meaningful?
Q10: Was appropriate statistical analysis used?

Risk of bias assessment of quasi-experimental studies

| Author | Q1 | Q2 | Q3 | Q4 | Q5 | Q6 | Q7 | Q8 | Q9 | Risk of bias |
| --- | --- | --- | --- | --- | --- | --- | --- | --- | --- | --- |
| Balci & Akdal, 2019 | Y | Y | NA | N | Y | Y | NA | Y | Y | **Low** |
| Çelebisoy et al., 2009 | Y | U | Y | Y | Y | Y | Y | N | Y | **Low** |
| Chang et al., 2008 | Y | Y | Y | Y | Y | Y | Y | Y | N | **Low** |
| Cohen-Shwartz et al., 2020 | Y | Y | Y | Y | Y | Y | Y | Y | Y | **Low** |
| D’silva et al., 2017 | Y | Y | Y | Y | Y | Y | Y | N | N | **Low** |
| Ganança et al., 2010 | Y | Y | NA | N | Y | Y | NA | N | N | **Moderate** |
| Jumani & Powell, 2017 | Y | Y | NA | N | Y | Y | NA | N | N | **Moderate** |
| Jung et al. 2012 | Y | Y | Y | Y | Y | Y | Y | N | N | **Low** |
| Kollén et al., 2006 | Y | Y | NA | N | Y | Y | NA | N | N | **Moderate** |
| Lim et al., 2021 | Y | Y | NA | N | Y | Y | NA | N | N | **Moderate** |
| Ribeiro et al., 2017 | Y | Y | Y | Y | Y | Y | Y | Y | N | **Low** |
| S et al. 2014 | Y | Y | NA | N | Y | Y | NA | N | N | **Moderate** |
| Se To, Ajit Singh, and Whitney 2022 | Y | Y | Y | Y | Y | Y | Y | Y | N | **Low** |
| Silva et al., 2014 | Y | Y | NA | N | Y | Y | NA | U | N | **Moderate** |
| Vaz et al., 2013 | Y | Y | NA | N | Y | Y | NA | N | N | **Moderate** |

Abbreviations: Y, yes; N, No; NA, not applicable; U, unclear
Q1: Is it clear in the study what is the ‘cause’ and what is the ‘effect’?
Q2: Were the participants included in any comparison similar?
Q3: Were the participants included in any comparisons receiving similar treatment/care, other than the exposure or intervention of interest
Q4: Was there a control group?
Q5: Were there multiple measurements of the outcome both pre and post the intervention/ exposure?
Q6: Was follow-up complete, and if not, were differences between groups in terms of their follow up adequately described and analyzed?
Q7: Were the outcomes of participants included in any comparison measured in the same way?
Q8: Were outcomes measured in a reliable way?
Q9: Was appropriate statistical analysis used?
